# A novel therapeutic effect of statins on nephrogenic diabetes insipidus

**DOI:** 10.1111/jcmm.12422

**Published:** 2015-01-16

**Authors:** Leonilde Bonfrate, Giuseppe Procino, David Q-H Wang, Maria Svelto, Piero Portincasa

**Affiliations:** a Department of Biomedical Sciences and Human Oncology, Internal Medicine, University Medical SchoolBari, Italy; b Department of Biosciences, Biotechnologies and Biopharmaceutics, University of Bari Aldo MoroBari, Italy; c Division of Gastroenterology and Hepatology, Department of Internal Medicine, Saint Louis University School of MedicineSt. Louis, MO, USA

**Keywords:** apical membrane, aquaporin, cholesterol-lowering drugs, hypercholesterolaemia, HMG-CoA, kidney, nephrogenic diabetes insipidus, vasopressin, water channels

## Abstract

Statins competitively inhibit hepatic 3-hydroxy-3-methylglutaryl-coenzyme A reductase, resulting in reduced plasma total and low-density lipoprotein cholesterol levels. Recently, it has been shown that statins exert additional ‘pleiotropic’ effects by increasing expression levels of the membrane water channels aquaporin 2 (AQP2). AQP2 is localized mainly in the kidney and plays a critical role in determining cellular water content. This additional effect is independent of cholesterol homoeostasis, and depends on depletion of mevalonate-derived intermediates of sterol synthetic pathways, *i.e*. farnesylpyrophosphate and geranylgeranylpyrophosphate. By up-regulating the expression levels of AQP2, statins increase water reabsorption by the kidney, thus opening up a new avenue in treating patients with nephrogenic diabetes insipidus (NDI), a hereditary disease that yet lacks high-powered and limited side effects therapy. Aspects related to water balance determined by AQP2 in the kidney, as well as standard and novel therapeutic strategies of NDI are discussed.

- Introduction
- Water balance and AQP2 regulation by vasopressin
- Nephrogenic diabetes insipidus (NDI)
  –Hereditary NDI
  –Acquired NDI
- Vasopressin-independent signals regulating AQP2 trafficking and potential use for NDI treatment
- Current treatment of NDI and use of statins
  –Statins and AQP2
- Advantages and disadvantages of statins in the treatment of NDI
- Conclusions

## Introduction

Statins are the first-line recommended pharmacological therapy in patients with dyslipidemias and for both primary 1 and secondary 2 prevention of coronary heart disease 3–6 (Table1). Statins are widely used to reduce risks for atherosclerotic cardiovascular disease 7,8 and associated morbidity and mortality, by decreasing plasma total and low-density lipoprotein cholesterol (LDL-C) concentrations 9,10. Statins occupy part of the active binding site of 3-hydroxy-3-methylglutaryl-coenzyme A (HMG-CoA) 11 and inhibit its enzymatic activity, a key step leading to the reduction in cellular sterol pool. Expression levels of LDL receptors are increased by a compensatory mechanism, leading to increased hepatic LDL uptake and decreased plasma cholesterol 12,13. Statins decrease biliary cholesterol output by reducing availability of biliary cholesterol 14–16 in both healthy individuals and hypercholesterolaemic patients 14–17. Statins have also beneficial effects on the vascular wall by stabilizing the atherosclerotic plaques, ameliorating impaired endothelial function, and reducing vascular inflammation 18.

**Table 1:** Multiple effects of statins

Effect(s)	Underlying mechanism(s)
Orthodox effects	
Decreased plasma LDL cholesterol levels (30-63%) 11,205–207 Modest increase in plasma HDL-cholesterol (≈5%) Decreased plasma triglyceride concentration (20-40%) Decreased incidence of coronary heart disease (primary and secondary prevention) 208	Inhibition of HMG-CoA reductase, reduced intrahepatic cholesterol, enhanced rate of hepatic LDL receptor cycling, increased LDL receptor turnover, reduced VLDL production (*via* hepatic apoB secretion), decreased recovery rate of HMG-CoA reductase activity
Pleiotropic effects	
Established (atherosclerotic diseases)	
Improved endothelial dysfunction 209	Increase of nitric oxide synthesis Improvement of blood flow dependent upon endothelium
Significant reduction of inflammatory markers (CRP) 210,211	Decreased monocyte expression of IL-6 and tumour necrosis factor-alpha or by direct suppression of CRP gene transcription 212
Decreased plaque growth 211	Decreased synthesis of extracellular matrix and proteins Rac1, RhoA
Stimulation of angiogenesis 213	Activation of protein kinase Akt in endothelial cells and by increasing the level of angiopoetine
Decreased plaque rupture or fissuration 214	Reduced metalloproteinases activity (MMP1, MMP3)
Prevention of thrombosis 215	Decrease in global fibrinolytic activity of the blood, decreased action of PAI-1 (and inhibition of thrombin generation
Potential (non-atherosclerotic diseases)	
Prevention of dementia 216,217	Reduced intracellular and extracellular levels of amyloid peptides; indirect effect *via* decreasing the risk of stroke
Preserved renal function 174,218	Improved vessel stiffening and endothelial function Reduced albuminuria
Improved bone metabolism 219–221	Increased bone formation through promotion of osteogenesis; Reduced risk of osteoporotic fractures, particularly in older patients
Improved outcome in chronic obstructive pulmonary disease (COPD) 222,223	Suppression of lung inflammation through inhibition of guanosine triphosphatase and nuclear factor-κB mediated activation of inflammatory and matrix remodelling pathways
Improved erectile dysfunction 224,225	Increased bioavailability of nitric oxide, enhanced plasma nitrite/nitrate concentrations and normalized RhoA and ROCK2 overexpression in corpora cavernosa
Prevention of gallstone diseases 226,227	Suppression of biliary cholesterol secretion and saturation, unrelated to modulation of cholesterol synthesis; inhibition of biliary cholesterol crystallization
Increased expression of AQP2 in the apical membrane of the kidney collecting duct principal cells [146 ] (see text and Fig.3 for details)	Reduced clathrin-mediated endocytosis and increased exocytosis; actin cytoskeletal reorganization through influence on Rho GTPases; facilitation of AQP2 insertion into the plasma membrane during VP/PKA/cAMP-induced AQP2 translocation

A recently identified ‘pleiotropic’ effect of statins is the increased expression levels of the renal membrane water channels Aquaporin 2 (AQP2). This effect is independent of classical cholesterol homoeostasis 19,20, but rather depends on depletion of mevalonate-derived intermediates of sterol synthetic pathways, *i.e*. isoprenoid intermediates, including farnesylpyrophosphate (FPP) and geranylgeranylpyrophosphate (GGPP).

This review will summarize aspects related to water balance, renal AQP2, vasopressin and nephrogenic diabetes insipidus (NDI), as well as current treatment of NDI and possible use of statins with respect to AQP2 trafficking.

## Water balance and AQP2 regulation by vasopressin

Water balance results from the equilibrium between daily water intake and urine excretion, in accord with daily changes of body and environmental factors 21. The kidney has a central role in preserving water balance: hypovolaemia and increased plasma osmolality stimulate aortic/carotid baroreceptors and hypothalamic osmoreceptors, respectively, to promote antidiuresis. The hypothalamus subsequently stimulates secretion of antidiuretic peptide hormone arginine vasopressin (AVP) from the pituitary gland. The terminal renal tubules at the level of connecting tubules and collecting ducts are characterized by variable permeability to water which is regulated by AVP and its interaction with the type 2 vasopressin receptor (AVPR2). The ultimate step in water reabsorption in the kidney is regulated by the interactions among AVP, AVPR2 and specific water channels, namely aquaporins (AQPs), playing critical roles in determining the cellular water content, and water balance in the body (See also http://www.nobelprize.org/nobel_prizes/chemistry/laureates/2003/) (access 09, 2014) 22–26.

**Fig 1:**
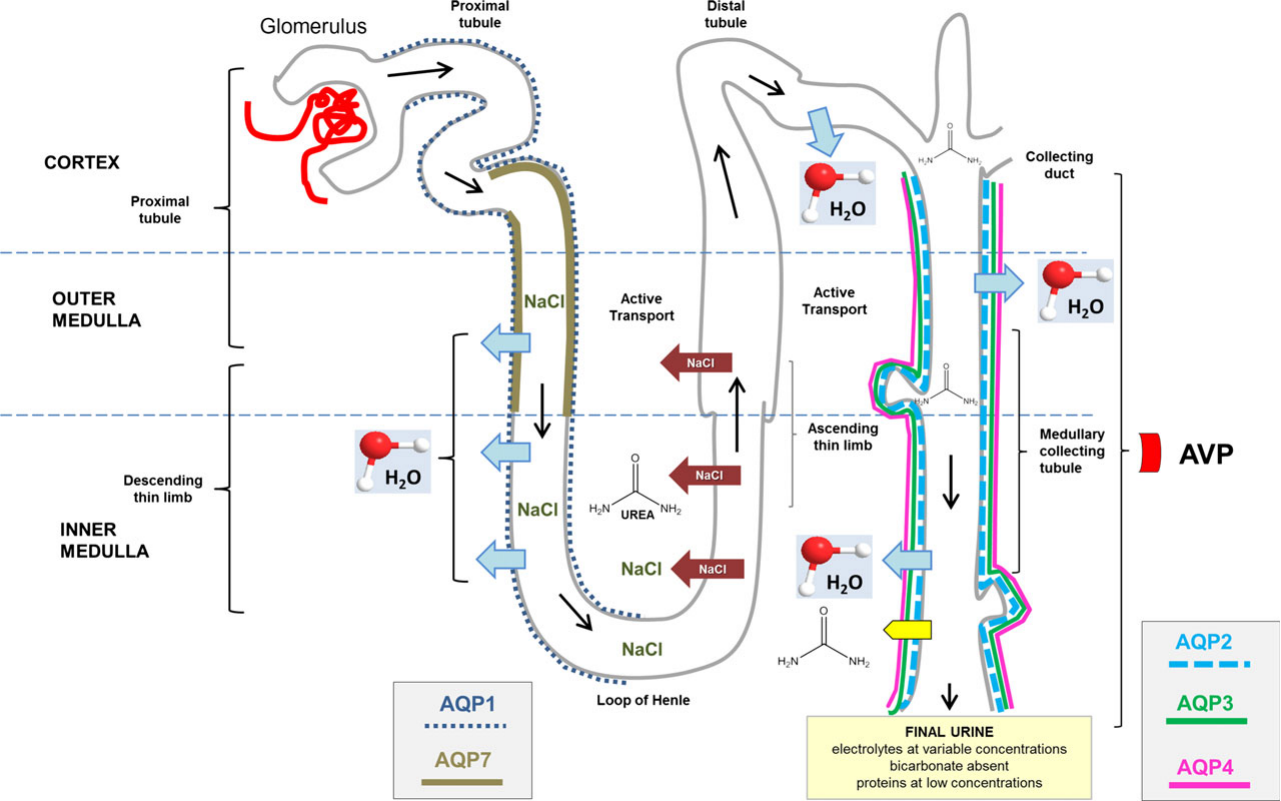
Anatomic structure of the nephron and collecting duct system, and localization of different aquaporins (AQPs) in the kidneys with vasopressin (AVP) effect. Sites of reabsorption of water and sodium chloride (NaCl) are shown. AQP6 is localized in the intracellular vesicle membranes of type-A intercalated cells of the collecting duct.

Aquaporins are widely distributed in all kingdoms of life from bacteria to plants and to mammals 27. There are 13 known mammalian AQPs, nine expressed in the kidney (AQP1, 2, 3, 4, 5, 6, 7, 8 and 11) 28–32. AQP1, 2, 3, 4 are involved in water transport across the epithelia of the renal tubule 33,34 (Fig.1). The role of AQP5 in type-B intercalated cells is still being investigated 35. AQP1 is found in proximal tubules and descending thin limbs of kidney. AQP2 is localized predominately in the intracellular vesicles and the apical plasma membrane of connecting tubule cells and collecting duct cells, while AQP3 and AQP4 are expressed in the basolateral plasma membrane of these cells 36–40. Water permeability and osmotic transport in the renal collecting duct depends upon the amount of active AQP2 (the principal AVP-sensitive water channel) in the apical plasma membrane of collecting duct principal cells. AQP2, normally stored in the cytosol 41,42 during diuresis, is re-directed and fused to the apical membrane of collecting duct principal cells following AVP stimulation 43,44. As homotetramer, AQP2 initiates water reabsorption within a favourable osmotic gradient between the lumen of the tubule and the interstitium. Electron microscopy studies confirmed the presence of intramembranous particle aggregates associated with enhanced water permeability 44,45. Intracellular movement of water is followed by rapid flux of water towards the basolateral membrane of collecting duct principal cells. After AVP stimulation has subsided, AQP2 water channels are removed from the apical membrane and returned to the cytoplasm by endocytosis 44,45.

**Fig 2:**
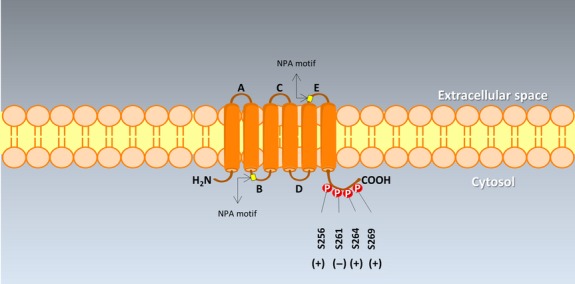
The topology of AQP2 with the COOH-terminal phosphorylation sites. AQP2 is a tetramer consisting of four identical protein subunits placed in the plasma membrane. Six transmembrane α-helices are arranged in a right-handed bundle and are represented by cylinders, with the amino (NH2-) and the carboxyl (COOH-) termini located on the cytoplasmic surface of the membrane. Five interhelical loop regions (A–E) form the extracellular and cytoplasmic vestibules. Loops B and E are hydrophobic loops that contain the highly, although not completely conserved, asparagine–proline–alanine (NPA) motifs. Such motifs appear to dip and overlap into the membrane, to construct the water pore 33,90. Serine residues at potential phosphorylation sites are labelled with their amino acid numbers at the carboxyl-terminal tail. AVP mediated increased (+) phosphorylation at S256, S264 and S269, and decreased (−) phosphorylation at S261. Both S269 and S256 phosphorylation are involved in AQP2 accumulation in the plasma membrane 50,246,247.

**Fig 3:**
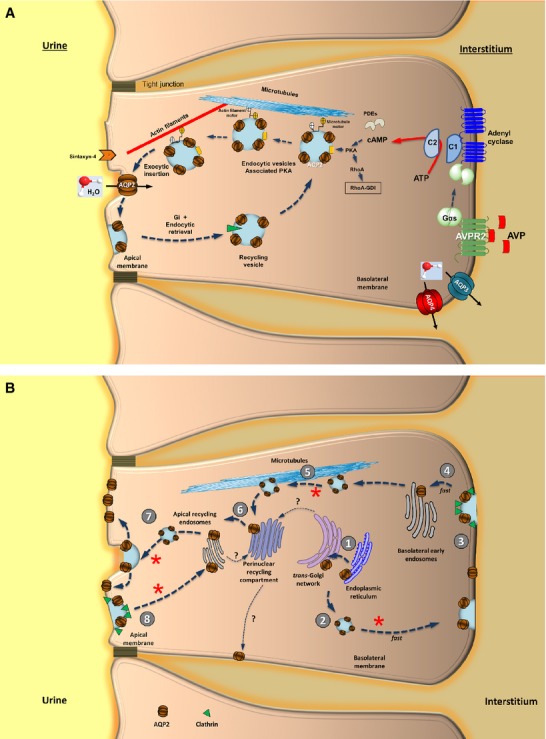
Molecular pathways involved in AQP2-mediated water transport in the kidney. (A) Signalling cascades and molecular pathways involved in AQP2-mediated water transport in relation to vasopressin (AVP) and vasopressin receptor (AVPR2) in the principal cells of the collecting ducts 22,33,37,115. The increased influx of water by AQP2 tetramer at the apical site requires a complex cascade of intracellular processes in concert with efflux of water by AQP3 and AQP4 tetramers at the basolateral membrane. The AVPR2 is composed of 7 membrane-spanning helices. Upon binding of AVP within the transmembrane helices II–IV, allosteric structural changes occur 78,79, the G-alpha-s heterodimeric protein is stimulated, and activates the adenylyl cyclase. This step results in increased intracellular levels of cyclic adenosine monophosphate (cAMP), activation of protein kinase A (PKA), phosphorylation of AQP2 in intracellular vesicles at serine 256 and other residues in the AQP2 OOH terminal 49,50 (see also Fig.2), trafficking of endocytic vesicles to the apical plasma membrane, and fusion of AQP2-containing vesicles with the apical membrane. As stated in the text, PKA is also responsible for phosphorylation of the membrane-associated RhoA, association with GDI to form the inactive complex RhoA-GDI, a step facilitating AQP2 insertion into the plasma membrane during VP/PKA/cAMP-induced AQP2 translocation 62. The docking system for vesicles might include specific receptors in the collecting duct cells which are associated with certain membrane domains housing AQP2 (*e.g*. syntaxin-4). Abbreviation: PDEs, phosphodiesterases. See also 33,37,247,248. (B) Proposed model of transcytotic trafficking of AQP2 from basolateral to apical membrane in principal cell of the collecting ducts. At least eight steps are involved: (1) Synthesis in the endoplasmic reticulum and transport to the *trans*-Golgi network; (2) rapid insertion of AQP2 into the basolateral membrane; (3) rapid internalization by clathrin-dependent endocytosis which is responsible for limited expression of basolateral AQP2. This step is blockable by low temperature (4°C); (5) AQP2 transcytosis to the perinuclear recycling compartment and the apical recycling endosomes *via* the microtubule-dependent mechanism. This step is inhibitable by colchicine; (7) exocytosis of AQP2 at the apical membrane; (8) recycling of AQP2 towards the apical recycling endosomes *via* the clathrin-dependent endocytosis. Thin dotted arrows show alternative pathways (?) of AQP2. Asterisks indicate where vasopressin (AVP) stimulus is inducing increased exocytosis and recycling of AQP2 with effect on transepithelial water flux (apical side) and cell migration, tubulogenesis, and likely transepithelial water flux (basolateral side). See also 69,70.

Binding of AVP (the polypeptide originating from the hypothalamus and migrating to the posterior pituitary through the supraopticohypophyseal tract 46) to AVPR2 results in COOH-terminal phosphorylation of the AVPR2. β-arrestin recruitment is followed by AVPR2 internalization, which implies the negative regulation of AVPR2 47. Upon AVPR2 activation, however, the signalling sequence involves Gsα dissociation, adenylyl cyclase activation, increased intracellular cAMP, activation of protein kinase type A (PKA), and phosphorylation of AQP2 at serine 256 plus other residues in the COOH terminus 48–50 (Fig.2). Thus, AQP2-bearing vesicles translocation to the plasma membrane is a combined effect of exocytosis and endocytosis 41,51–55 (Fig.3A and B). The process of intracellular vesicular trafficking is complex and requires several proteins. G proteins and subunits G_1_ and G_0_ assist exocytosis and endocytosis and heterotrimeric G proteins from the Gi family are involved in cAMP-dependent trafficking of AQP2 26. Monomeric GTP-binding proteins belonging to the Rab family also play a key role in the context of intracellular vesicle trafficking of AQP2 56. The Ras superfamily of small GTP-binding proteins is also involved in vesicle trafficking and regulates actin cytoskeleton organization and actin polymerization 57. Activation of proteins of the Rho-family occurs: Rac1 (formation of lamellipodia), Rho (formation of actin-based structures of filopodia, regulation of stress fibres and formation of focal adhesion complexes 58) and Cdc42 (activator of Rac1 and Rho). GTP-binding proteins from the Rho-family fluctuate from active GTP-bound status (when Rho is bound to its putative effectors, the Rho kinases 59) to inactive GDP-bound form; this interconversion is regulated by factors including GEP (GDP/GTP exchange protein), GAP (GTPase activating protein, which binds to the GTP-form and stimulates the intrinsic GTPase activity of monomeric G proteins) and GDI (GDP dissociation inhibitor which inhibits GDP dissociation, prevents GTP hydrolysis and maintains the Rho-family members in a soluble form) 60,61. In particular, translocating the membrane-associated active Rho form to a soluble compartment implies inactivation *via* Rho-GDI interaction. Decreasing Rho activity implies depolymerization of F-actin, which is considered a physical barrier preventing AQP2-containing vesicles exocytosis, and greater insertion of AQP2 into the apical plasma membrane 62. This step is clearly shown for RhoA, following phosphorylation by PKA at Serine 188 63, a regulatory mechanism also operating in the case of AQP2 trafficking (see below and Table2) 62. A short-term regulation (5–15 min.), mainly dependent on AVP 51, is the one which affects the trafficking of AQP2-containing membrane vesicles to and from the apical membrane. The long-term regulation (>24 hrs) of renal water permeability implies the overall effect on *AQP2* gene and AQP2 protein abundance in the cell, also under the AVP control 43,54,64. In the latter case, dysregulation of such mechanisms is responsible for clinical conditions characterized by disturbed water balance (Table3). Furthermore, AQP2 recycles constitutively between cell surface and intracellular vesicles, independently of AVP stimulation 65–67.

**Table 2:** Pathways involved in AQP2 trafficking in the kidney

Pathway	Mechanism(s)
Activation of the G-coupled V2 receptor 21,26,43,49,51–54,64,68,71,74,83	AVP-dependent cAMP/PKA activation Phosphorylation of AQP2 at Ser 256 Redistribution of AQP2 to the plasma membrane
Nitric oxide/cGMP pathway 48,128,130,131,228	Effect of phosphodiesterase inhibitors, sodium nitroprusside and L-arginine
COX/prostaglandin E_2_ pathway 134,229,230,155	Effect of cyclooxygenase (Cox) 2 inhibitors Effect of EP2 and EP4 receptors agonists
Modulation of actin cytoskeleton network	
Statin-mediated 49,151,228	Inhibition of conversion of HMG-CoA to mevalonate Decreased prenylation and consequent down-regulation of RhoA GTPases (fast) Plasma membrane depletion in cholesterol (slow) Inhibition of AQP2 endocytosis
Statin-independent 132,148,149,151–153	Plasma membrane depletion in cholesterol (*e.g*. methyl-beta-cyclodextrin) and inhibition of clathrin-mediated AQP2 endocytosis
	Phosphorylation of RhoA by PKA, reduced RhoA membrane association, increased AQP2 translocation 42,154

**Table 3:** Disorders of water balance associated with dysregulation of AQP2

Disorder	Description
Polyuric syndromes	
Central diabetes insipidus Compulsive water drinking Cultural overhydration	Associated with low levels circulating vasopressin and decreased amount of AQP2 in collecting duct cells 115
Nephrogenic diabetes insipidus (NDI) ∘Heritable X-linked NDI (mutation of the *V2R* receptor gene) ∘Acquired NDI in case of sustained: ureteral obstruction hypokalaemia hypercalcemia lithium intake, other drugs inflammation	Polyuria associated with depletion of renal AQP2 protein from the collecting ducts and connecting tubules
∘Autosomal dominant/recessive (mutation in the *AQP2* gene)	Impaired trafficking of AQP2 Lack of fusion with the apical membrane and/or Decreased channel function
Extracellular fluid volume (ECF)-expanded states Congestive heart failure Hepatic cirrhosis Nephrotic syndrome	Oedematous disorders 231

AQP, aquaporin; NDI, nephrogenic diabetes insipidus.

Aquaporin 2 is constitutively targeted to the basolateral membrane in canine polarized (MDCK)- kidney cells, and is retrieved by clathrin-mediated endocytosis into Rab5-positive vesicles. The microtubule-dependent (colchicine-sensitive) transcytosis of AQP2 might involve intracellular organelles, *i.e*. the endoplasmic reticulum, *trans-*Golgi network, perinuclear recycling compartment, and apical recycling endosomes within Rab11-positive vesicles (for continuous recycling between the apical membrane and the perinuclear region). Thus, a novel role for AQP2 has been suggested, *i.e*. cell migration, tubulogenesis, epithelial morphogenesis and, possibly, transepithelial water flux 68–70 (Fig.3A and B). AVP, aldosterone and hypertonicity also enhance AQP2 expression at the basolateral membrane, as shown by both *in vitro* and *in vivo* studies 71–73. Moreover, AVP leads to increased urine osmolality to about 1200 mOsm/kg with decreased urine output to 0.5 ml/min. The opposite is seen without AVP (*i.e*. urine osmolality decreased to about 50 mOsmol/kg and urine flow rate to 20 ml/min.) 74.

## Nephrogenic diabetes insipidus

Diabetes insipidus is characterized by polyuria and compensatory polydipsia and encompasses four types: (*i*) the central form (the congenital familial neurohypophyseal diabetes insipidus or the acquired form); (*ii*) the NDI; (*iii*) the gestational diabetes insipidus; and (*iv*) the primary polydipsia (see review 21 for details). NDI is a syndrome in which the kidneys fail to conserve water because of variable degrees of resistance to AVP and can be hereditary or acquired.

### Hereditary NDI

The hereditary NDI is a rare disorder appearing in infancy characterized by resistance to ADH, polyuria and polydipsia 75,76. This disease is caused by mutations in either the *AVPR2* or *AQP2* genes 37,75,77–79. About 90% of the patients with congenital NDI are diagnosed because of the presence of *AVPR2* gene mutations (X-chromosome at Xq28) 80 leading to a dysfunctional AVPR2. Over 220 mutations have been identified so far, including missense/nonsense, splicing, small deletions, small insertions, small indels, gross deletions, gross insertions/duplications and complex rearrangements. Mutations in *L1CAM*, a gene close to the *AVPR2* gene, may also account for some rare cases of NDI 21,81 (refer to http://www.ndif.org) (access 09, 2014). Five classes of *AVPR2* gene mutations have been described 82 and comprise: a truncated receptor protein, a misfolded receptor (retained in the endoplasmic reticulum), a receptor unable to elicit cAMP production or to interact with AVP at the cell surface, and a receptor protein misrouted to intracellular organelles. Mouse models have been produced for X-NDI to better understand compensatory changes in the kidney and innovative treatments 83–85. Mild phenotypes of NDI have been also identified and are consistent with a number of additional mutations (*e.g*. p.Arg104Cys or p.Ser329Arg) 21. The X-linked inheritance implies that more pronounced polyuria is observed in males. Patients do not improve even after administration of exogenous AVP 86. The defect is present at birth with significant variability because of partial or incomplete NDI; patients have large volumes (more than 30 ml/kg/day, *i.e*. >3 l/day in adults or >2 l/m^2^ in children) of dilute urine (less than 250 mOsmol/kg) produced and associated with exaggerated thirst. Thus, typical symptoms of NDI include polydipsia, polyuria, hypernatremia and dehydration 37,51. Hypernatremia is usually associated with reduced feeding and weight loss, irritability, dry skin, and recessed eyeballs 87. Potential long-term complications of NDI are mental retardation, megacystis, hydroureter, hydronephrosis and renal failure 87–89.

Another hereditable form of NDI involves the autosomal recessive or the autosomal dominant forms conferring the mutations in the *AQP2* gene on chromosome 12q13 encoding a 271-amino acid protein 33,55,90. More than 50 mutations in the *AQP2* gene have been described, so far, including missense/nonsense, splicing, small deletions and small insertions. The mutations imply decreased channel function and/or defective fusion of the AQP2 (retained in the intracellular space) 91 with the apical membrane 42,92. The autosomal recessive NDI is seen in patients who are homozygous or compound heterozygous for mutations in the *AQP2* gene. As a result, abnormalities consist of AQP2 misfolding, retention in the endoplasmic reticulum, or rapid degradation of the water channel protein 82. This NDI variant is encountered equally in both genders and starts at birth with a severe clinical picture, although partial NDI is rarely seen 74. The dominant form of NDI accounts for 10% of autosomal cases, and is because of the mutations involving the carboxyl tail of AQP2 and therefore the water channel intracellular routing 21. Abnormalities include AQP2 misrouting 93, intra-Golgi retention, or routing of AQP2 to lysosomes, late endosomes, or basolateral plasma membrane, where AQP3 an AQP4 should be, instead 94.

### Acquired NDI

The acquired NDI syndromes are the commonest clinical conditions (Table3). All forms are characterized by decreased expression of AQP2 or abnormal trafficking of AQP2 to the apical plasma membrane. Reduced expression of AQP2 is encountered in both acute and chronic renal failure 21. Either bilateral or monolateral sustained ureteral obstruction is associated with persistently decreased AQP2 mRNA and protein levels in the inner medullary collecting ducts 95,96. Abnormal transcriptional pathways or regulation of mRNA degradation might be involved 96, as AQP2 trafficking to the apical plasma membrane of collecting duct principal cells is still functional after ureteral obstruction 95–97. The vasopressin receptor or its coupling to adenylyl cyclase also appears to be affected by the obstruction 98. During ureteral obstruction, a role for intrarenal angiotensin II generation in inhibiting vasopressin signalling and cyclooxygenase-2 (COX-2) in impairing renal handling of sodium and water has been advocated. Pharmacological manipulation with angiotensin receptor blockers (*e.g*. candesartan) 99 or COX-2 inhibitors 100,101 might prevent the reduction in AQP2 down-regulation and post-obstructive polyuria, as seen in animal models of ureteral obstruction.

Following treatment with lithium salts in bipolar affective disorders, up to 40% of patients may develop lithium-induced NDI 102–104. In rats, long-term treatment of lithium is associated with >90% decrease of AQP2 protein levels in the kidneys and severe polyuria, partly reversible 105. Decreased AQP2 mRNA abundance has been advocated to explain reduced AQP2 protein levels 106. The effects of lithium on the kidneys impact the calcium-sensing receptor and the calmodulin-dependent pathways 107,108, and COX-2 function 109. Some proteins involved in a myriad of functions, *i.e*. regulation of gene expression, signal transduction, cytoskeletal organization, cellular reorganization, cell proliferation and apoptosis, might also be affected 21,110. Antibiotics (*e.g*. demeclocycline 111 and foscarnet 112), antifungals (*e.g*. amphotericin B 113), and antineoplastic drugs (*e.g*. ifosfamide 114) might also cause reversible forms of acquired NDI. Hypokalaemia-induced NDI with polyuria and defective urinary concentrating ability may follow inappropriate diuretic therapy or primary aldosteronism 115. Central mechanisms in the brain might be also involved (*e.g*. inhibition of vasopressin secretion 116, or primary polydipsia 117). Reduced AQP2 expression levels in the inner medulla and cortex and decreased urinary concentrating capacity are found in rats following a potassium-deficient diet 118, and could follow an early (12–24 hrs) hypokalaemic effect on AQP2 protein and mRNA concentrations 119. Hypercalcaemia is also associated with decreased AQP2 expression and the mechanism is likely mediated by hypercalciuria, the calcium-sensing receptor and calcium-dependent activation of the proteolytic enzyme calpain 108,120,121. Inflammatory conditions are associated with polyuria and impaired renal concentrating ability, as shown in dogs and cats with pyometra 122,123. This is likely because of activation of inflammatory cytokine signalling pathways resulting in decreased expression levels of AQP2 and V2 receptors in the renal medulla 124. Also, NF-*k*B, interleukin-1β, and bacterial species (*Escherichia coli*, *Klebsiella*)-dependent endotoxins might influence *AQP2* gene expression, vasopressin binding, vasopressin V2 receptors, and AQP2 protein concentrations 124–127.

## Vasopressin-independent signals regulating AQP2 trafficking and potential use for NDI treatment

In 90% of the cases, NDI is transmitted as an X-linked recessive trait caused by mutations in the *V2R* gene. To rescue the inactivation of the V2R-elicited cAMP pathway, alternative intracellular pathways might be activated, which promotes AQP2 trafficking towards the plasma membrane. Different intracellular pathways appear to be involved in regulating AQP2 translocation (Table2), besides the classical regulation which is mediated by the specific G protein-coupled AVPR2 21.

Arginine vasopressin-independent pathways could lead to AQP2 expression at the plasma membrane in renal cells. The nitric oxide/cGMP pathway is one of the most interesting pathways 128 and implies the formation of nitric oxide from L-arginine, activation of soluble guanylate cyclase (GC), and increased intracellular cGMP concentration. Activated PKG can phosphorylate AQP2 directly or indirectly through PKA activation 63. Indeed, mice lacking all the nitric oxide synthase isoforms developed NDI 129. Moreover, the cGMP phosphodiesterase inhibitor sildenafil (Viagra), increased insertion of AQP2 in the apical membrane of renal cells both *in vivo* and *in vitro* 130 and reduced polyuria in rats with lithium-induced NDI 131. Prostaglandins, in particular E2 (PGE2), are abundantly expressed in the kidney and are considered modulators of AQP2 plasma membrane expression. The EP1-4 receptors have the 4 receptor subtypes through which PGE2 exerts its pharmacological actions 132,133. EP1 receptors preferentially couple to an increase in cell calcium. EP2 and EP4 receptors stimulate cyclic AMP through a Gs subunit, whereas the EP3 receptor preferentially couples to Gi, inhibiting cyclic AMP generation. COX inhibitors decrease PGE2 production and counteract the inhibitory role of EP3 receptor on cAMP production, thus increasing AQP2 exocytosis. Pharmacological stimulation of EP2 and EP4 alleviates NDI in the mouse and rat experimental models of the disease 84,134. With a similar mechanism calcitonin, the hormone produced by parafollicular cells, increases AQP2 apical targeting *in vitro* and *in vivo* by activating its Gs-coupled cognate receptor expressed in collecting duct renal cells and markedly ameliorates polyuria in vasopressin-deficient Brattleboro rats 135.

A therapeutic approach based on one of the molecules listed above might achieve a positive clinical outcome in patients affected by NDI.

### Current treatment of NDI and use of statins

Exogenous administration of the AVP analogue desmopressin is used to treat central diabetes insipidus 136 and nocturnal enuresis 137. This approach, however, is ineffective in patients with congenital NDI because mutations in the V2R or AQP2 genes inactivate these proteins.

Gene therapy to cure NDI remains experimental and highly speculative 138. Acquired NDI may benefit from treatment of the underlying condition, and revision of dosage/discontinuation of an inciting drug. Treatment of hereditary NDI, however, remains a significant challenge, mainly because of the lack of function of AVPR2 and the lack of effect by desmopressin (Table4). To prevent severe complications, treatment of congenital NDI must start in infancy; high doses of desmopressin may be effective in patients with partial NDI or in heterozygous females with polyuria, when some AVPR2 function is retained. In the other cases, water intake must be appropriate to counteract water loss causing polydipsia and polyuria. The quality of life, however, is negatively affected by excessive drinking and urination and by potential complications. Low sodium diet and drugs such as diuretics and NSAIDs might have additional benefits (*i.e*. increased urine osmolality and 30–70% decrease of urine volume) 139–141.

**Table 4:** Standard and experimental therapeutic approaches to hereditary nephrogenic diabetes insipidus

Regimen	Notes
Standard Infants: minimizing polyuria, preventing hyponatremia and volume depletion Adults: correcting underlying disorder Continuous water intake (every 2 hrs, day and night) Prevent hydronephrosis and bladder dilatation/dysfunction	Inability to respond to increased thirst Instruct to frequent/double voiding
Low salt (≤2.3 g sodium/day), low protein (≤1 g/kg/day)	Decreased dietary solute and urine output 87. Difficult to maintain o a long-term basis.
Diuretics (thiazide, amiloride) 142,232,233	Block of Na-Cl cotransporter in the distal tubule (thiazide) and of the Na channel EnaC in the connecting tube (amiloride) resulting in decreased sodium and water reabsorption, and hypovolaemia Activation of the renin-angiotensin II-aldosterone system, increased sodium reabsorption (proximal tubule) and AQP1-dependent increase in water reabsorption, with relieve for the AQP-2 dependent water absorption (distal tubule and collecting duct) Association with amiloride leads to additional beneficial effects Amiloride-dependent increase in AQP2 levels (?) 234 Mild electrolyte complications possible
NSAIDs (indomethacin more effective than ibuprofen) 87,145	Inhibition of renal prostaglandin synthesis and decreased antagonism of ADH. Increased concentrating ability 235,236 Potential side effects as a result of long-term treatment
Experimental AVPR2 chaperones	Promotion of intracellular proper maturation, folding of AVPR2 receptor followed by expression of a functional cell surface AVPR2 237–240 Unspecific chemical chaperones (poor outcome): glycerol, DMSO 241 Peptide pharmacochaperones: cell-permeable AVPR2 antagonists 242 (need for being released by the receptor), AVPR2 agonists 240 Nonpeptide pharmacochaperones: antagonist (see review 21) and agonists (initiate cAMP response 242). Effect dependent on the type of AVPR2 mutation, possible interaction with other receptors, competitive effect with AVP.
AQP2 water channel chaperones	Molecules helping to direct intracellularly retained AQP2 to cell surface 91,243 (research in progress)
AVPR2 bypass (Increased trafficking, abundance and phosphorylation of AQP2 to the cell membrane of collecting tubule cells) 84,134,228	Statins: effect independent of AVP, AVPR2, and cAMP (see text for details) 49,148,228 cGMP pathway activation: L-arginine, sodium nitroprusside, atrial natriuretic peptide 128,228, phosphodiesterase (PDE5) inhibitor sildenafil citrate (Viagra) (?) 130 cAMP pathway activation: phosphodiesterase (PDE4) inhibitor rolipram (?) 244, calcitonin (*via* GαS-mediated intracellular increase of cAMP 135,245 Prostaglandins: acting as specific E-prostanoid-receptor agonists (EP2, EP4). Decrease AQP2 internalization 85,134 (*e.g*. ONO-AE329) 84, butaprost, CAY10580 134 Heat shock protein 90-inhibitor (17-allylamino-17-demethoxygeldanamycin) (?): might induce proper folding of AQP2 retained in the endoplasmic reticulum 241,243

DMSO, dimethylsulfoxide; EP, prostaglandin E; NSAIDS, non-steroidal anti-inflammatory drugs.

Urine output could be reduced by ∽70% when hydrochlorothiazide diuretic (25 mg daily) is used with very low sodium-restricted diet of 9 mEq/day 142. Potassium sparing agents such as amiloride, might have an additive effect with thiazide diuretics, *via* mechanisms likely including the inhibition of potassium loss induced by thiazides 143. Diuretics in NDI are likely to reduce urine output by promoting proximal reabsorption of sodium and water. In this condition, less water is delivered to the AVP-sensitive tract of the nephron, the collecting duct.

Renal prostaglandin synthesis (mediated by the prostaglandin synthetase) is inhibited by NSAIDs. The effect of NSAIDs in NDI is based on the inhibition of the antagonizing effect of prostaglandins on AVP. A better urinary concentration is achieved with NSAIDs, and output in NDI can be reduced by 25–50% 144,145.

Because of poor therapeutic outcome and potential persistently severe side effects (*e.g*. renal and gastrointestinal complications), the attention has moved from the above-mentioned therapeutic regimens to novel strategies (Table4). Statins, the cholesterol-lowering agents acting on HMG-CoA reductase, have promising effects by working on mechanisms totally different from AVP and cAMP.

### Statins and AQP2

Recent investigations have shown that statins increase AQP2 expression in the apical membrane of the collecting duct principal cells in the kidneys 49,146–149. Early *in vitro* experiments on renal MCD4 cells have shown that long-term treatment (3 days) of lovastatin might do so by reducing plasma membrane cholesterol 147 (also see below). The same group reported that fluvastatin acts on mouse kidney collecting duct cells by a vasopressin-independent mechanism, and this effect leads to water retention, reduces urine volume, and increases urine osmolality in mice 148.

Li *et al*. 49 used cell cultures and *in vitro* kidney slice from Brattleboro rats to assess AQP2 trafficking in response to incubation with simvastatin. Short-term exposure to simvastatin produces no change in cholesterol plasma membrane levels, but increases AQP2 accumulation in the apical membrane of principal cells of kidney slices from Brattleboro rats. At variance with VP effect, the action of statins is not associated with increased intracellular cAMP or inhibited by the PKA inhibitor H-89. Instead, the mechanism of action of simvastatin appears to be independent from cAMP/PKA activation and the phosphorylation of AQP2 at Ser256 which represents the classical pathway of VP-regulated AQP2 trafficking (Fig.2). Mechanisms of decreased constitutive endocytosis and/or increased constitutive exocytosis of AQP2 might be also affected by statin treatment 147,148. Clathrin plays a major role in the formation of coated vesicles and is involved in endocytosis 67,66. Li *et al*. 49 showed that simvastatin induces membrane accumulation of AQP2 in LLCPK-1 cells because of reduced clathrin-mediated endocytosis, rather than increased exocytosis. Same effects on AQP2 endocytosis in MCD4 cells were shown in a parallel study by Procino *et al*. 121.

The statin-mediated inhibition of the early step in the cholesterol biosynthetic pathway in any targeted tissue (*i.e*. catalyzation of HMG-CoA to mevalonate), leads in turn to the inhibition of the synthesis of isoprenoid intermediates such as FPP and GGPP 13. FPP and GGPP act as lipid anchors required for membrane tethering and activation of several proteins, such as heterotrimeric G proteins and small GTP-binding proteins (in particular the family of Ras from FPP, and the families of Rho, Rap and Rab GTPases from GGPP). Finally, the effect of early inhibition of mevalonic acid synthesis will be the downstream inhibition of several intracellular signalling molecules, accounting for the so-called ‘pleiotropic effects’ of statins. This scenario also applies to AQP2 trafficking in the kidneys. The above-mentioned effect of statins on isoprenoid intermediates might partly explain the lack of posttranslational changes of several signalling proteins (*e.g*. small GTP-binding proteins), as such molecules assist a number of cellular functions including cytoskeletal assembly as well as trafficking of proteins and lipids 150.

A previous *in vitro* study 151 has found that the statin-mediated inhibition of isoprenylation of Rho GTPase decreases the endocytosis of fluorescein isothiocyanate (FITC)-labelled albumin in kidney cells. The activation of this pathway results in the actin cytoskeletal reorganization and plays a role in protein trafficking and intracellular transport processes. Moreover, statins influence Rho GTPases which regulate the cytoskeleton 152,153. Both elements likely regulate vesicle trafficking and endocytosis 154,155.

Procino *et al*. 148, demonstrated that both fluvastatin and isoprenylation inhibitors significantly reduced the amount of active membrane-tethered RhoA and Rab5 GTPases with a parallel increase of AQP2 plasma membrane expression *in vivo* and *in vitro*. The study of Li *et al*. 49 confirmed that the clathrin-dependent effect of statins on AQP2 endocytosis involves the down-regulation of Rho GTPase (specifically RhoA) activity in a dose-dependent manner, and is already evident at concentrations as low as 10 μM. In particular, simvastatin-dependent accumulation of AQP2 at the plasma membrane could be prevented in transfected cells by overexpressing the constitutively active RhoA G14V, but not by the dominant negative RhoA T19N. They concluded that, among the family of Rho GTPases, RhoA is involved in simvastatin-mediated membrane trafficking of AQP2 49.

In wild-type C57BL/6 mice intraperitoneal injection of different classes of statins showed that fluvastatin was as effective as AVP in promoting AQP2 apical accumulation in the kidney collecting ducts 148. In the same work, prolonged treatment of fluvastatin induced a significant reduction of the diuresis and increase of urine osmolality with no effect on glomerular filtration rate 148.

Brattleboro rats lacking AVP because of spontaneous mutation of the *AVP* gene 66,156, were treated with intraperitoneal administration of simvastatin to a final plasma concentration of 200 μM, without any visible side effect 49. Simvastatin caused a decrease in urinary volume associated with consistently increased urine osmolality. Immunofluorescence staining of AQP2 revealed a significant increase in the apical membrane of the principal cells of the collecting duct in the cortex and outer medulla of the kidney of simvastatin-treated animals. More recently, it has been shown that a administration of a combination of secretin and fluvastatin dramatically reduced the polyuria and increased urine osmolality in the mouse model of X-linked NDI 149.

It is unclear whether additional membrane transporters might be influenced by statins, inducing AQP2 trafficking 146. Subcellular distribution of A and B subunits of V-ATPase, a protein showing membrane recycling, is not affected by simvastatin 49. In the study by Procino *et al*. 148, additional basolateral and apical membrane Na^+^ transporters (Na^+^/K^+^-ATPase and NKCC2) were up-regulated in the kidney membrane fraction by fluvastatin, suggesting that these transporters might contribute to Na^+^ and water reabsorption.

The statin-dependent inhibition of isoprenylation might affect additional Rho GTPases (*e.g*. Rac1 and Cdc42) and lead to an acute effect on AQP2 trafficking 154,157. Li and colleagues 49 demonstrated an acute effect of statins (within 60 min.) after a single injection with disappearance in 5–6 hrs. Likely, the simvastatin-mediated effect would be rapid modulation of RhoA GTPase activity, rather than cholesterol depletion 158, since a longer time (more than 35 hrs) is required for statins to induce 50% depletion of cholesterol membrane and influence trafficking of proteins and vesicles 159,160.

Studies on the effect of statins on AQP2 trafficking in animal models 49,147–149 used statins doses that are commonly used in rat/murine studies 161–163. However, because of the rapid up-regulation of HMG-CoA reductase observed in rodents during statin treatment 164, these doses are higher than those used in humans. The doses used in these studies are not therefore predictive of those needed in humans to achieve the same result. In addition, personal unpublished observations from these authors indicate that administration of different statins doses increases AQP2 plasma membrane expression in patients requiring hypocholesterolaemic therapy. Therefore, statins doses in the range of the currently used to reduce blood cholesterol, might be beneficial for NDI patients.

Statin-independent mechanisms might also promote AQP2 accumulation at the plasma membrane of kidney cells. For example, decreasing plasma membrane cholesterol by the cholesterol-depleting drug methyl-β-cyclodextrin (mβCD) 66,165, a blocker of clathrin-mediated endocytosis 160,166,167 including AQP2 67,66, is associated with rapid accumulation of AQP2 in cultured kidney epithelial cells and in principal cells of the intact perfused kidney. Furthermore, functioning of Rho-family GTPases (including RhoA) might follow an isoprenylation-independent pathway, *i.e*. phosphorylation of RhoA by PKA at Ser188. This step is a key event for cytoskeletal dynamics controlling cAMP-induced AQP2 translocation, and would lead to increased association with GDI (RhoA-GDI) and reduced RhoA membrane association and activity 168. The attenuation of Rho activity results in depolymerization of F-actin, facilitating AQP2 insertion into the plasma membrane during VP/PKA/cAMP-induced AQP2 translocation 62.

A different pathway leading to increased AQP2 abundance in the apical membrane might involve the nuclear receptor peroxisomal proliferator-activated receptor subtype γ (PPAR-γ). Indeed, the synthetic PPAR-γ agonist rosiglitazone, besides improving insulin resistance, is associated with fluid retention and oedema. This side effect appears to be mediated by an increase in sodium and water retention (*via* increased abundance of AQP2, and AQP3) in the kidney 169,170.

## Advantages and disadvantages of statins in the treatment of NDI

The effects of statins with respect to AQP2 trafficking and water reabsorption in the kidneys have been raising much interest about their potential therapeutic pleiotropic effects in patients with NDI. Pilot studies from our group suggest that simvastatin increases AQP2 plasma membrane expression in humans treated for hypercholesterolaemia. The dose effect of different statins, however, needs to be tested in clinical trials with respect to duration of treatment, pharmacokinetics and lipophilic properties of different molecules 171.

The possibility of adverse reactions during long-term use and high-dosage statin therapy needs to be addressed. This aspect is of interest in patients with NDI who will likely require chronic treatment of statins.

In healthy individuals, atorvastatin treatment leads to modest and transitory decrease in sodium excretion and no change in renal function. In the same study, no change was documented in glomerular filtration rate, vasoactive hormones, tubular function and renal plasma flow 172. Some statins (simvastatin or rosuvastatin 173), might induce tubular inhibition of small-molecular-weight proteins and transient low-molecular-weight proteinuria 151,174,175. Hyperlipidemic patients administered with rosuvastatin 10 or 20 mg/day for 3 months, for example, show a dose-dependent increase in urinary low-molecular protein α-1 microglobulin 176. A plausible explanation might be the inhibition of HMG-CoA reductase in the proximal tubule cells. This step leads to a depletion of the cellular geranylgeranyl pyrophosphate pool (an intermediate of the sterol pathway) and reduced function of one or more GTP-binding proteins, which are known to be involved in the process of proximal tubular endocytosis 151,177–180. There is evidence suggesting that increased transient low-molecular-weight proteinuria following statin treatment is rather a benign outcome 181. Renal failure has been rarely reported with high doses (80 mg/day) of rosuvastatin. Renal adverse events have also been reported with other statins 182–185. By contrast, patients taking statins often suffer from underlying chronic kidney disease and still, statins reduce proteinuria and glomerular filtration rate 186, without aggravating renal failure 187,188 or aggravating proteinuria 189. The use of statins is also advised to persons with chronic renal insufficiency 190.

A recent study investigating the short-term (13 days) effect of statins on the urinary protein concentration and proteome in healthy volunteers found that either rosuvastatin (40 mg/day) or pravastatin (80 mg/day) did not induce major changes in the urinary protein concentration/proteome (on a background of high variability in the baseline urinary proteome/proteins among volunteers 191). In the animal model, statins prevented the development of renal injury and enhanced renal perfusion 192,193. A simvastatin-dependent increase in nitric oxide mediated the amelioration of glomerular filtration rate, renal plasma flow and endothelial function in patients with autosomal dominant polycystic kidney disease 194. Improved renal function was observed in statin-treated patients with ischaemic heart disease 195. In patients with already impaired glomerular filtration rate, statins did not change or slightly increased urinary albumin excretion, independently on dose or type of statins 196.

Muscle injury ranging to myalgias (up to 10%) 197 even with normal creatine kinase concentration, to myositis (0.5%) to rhabdomyolysis (<0.1%) eventually evolving to acute renal failure from myoglobinuria have been reported in some patients using statins with a median time of 1 month. Pravastatin and fluvastatin have the lowest rate of muscle side effects. Statin-associated myopathy is enhanced in patients with decreased thyroid function, acute and chronic renal failure, and obstructive liver disease.

Statin-induced liver injury disclosed by mild persistent elevations in aminotransferases has been reported in up to 3% of patients receiving statins (1.2 episode/100,000 users), usually during the first 3 months in a dose-dependent fashion 198. The true importance of such possibility has been questioned by several studies comparing statin use with placebo or with the general population 199–202.

Forms of reversible cognitive dysfunction and memory loss have been associated particularly with lipophilic simvastatin, pravastatin and atorvastatin 203.

Reports have associated the use of some statins with the increased risk of developing diabetes mellitus in non-diabetics. In diabetic patients, furthermore, the glycaemic control might become more problematic with the intensive use of some statins 204.

Statin use must be also discontinued during pregnancy (increased risk of congenital central nervous system and limb abnormalities) and breastfeeding.

Whether longer treatment periods might change such outcome is currently unknown. Also, the ultimate interaction between statin-dependent proteinuria and AQP2 (as well as other kidney AQPs) will be the focus of further clinical research.

## Conclusions

The regulation of AQP2 expression in the kidney tubule is a key step in maintaining water balance. NDI represents a severe disturbance of water homoeostasis, exposing to polydipsia, polyuria, hypernatremia and dehydration. A better knowledge about NDI has recently emerged with genetic, clinical, molecular and pathophysiological perspectives. Statins improve cardiovascular outcome, and evidence shows that statins modulate the expression of AQP2 mRNA and protein in the kidneys, thereby increasing water reabsorption. This non-lipid dependent pleiotropic property of statins, if proven to be effective and well-tolerated, will open new venues to the treatment of hereditable NDI. It is possible that the beneficial effects of statins on NDI will outweigh the overall limited risk of adverse effects.
